# Implications of Foliar Particulate Matter Deposition on the Physiology and Nutrient Allocation of Dominant Perennial Species of the Indo-Gangetic Plains

**DOI:** 10.3389/fpls.2022.939950

**Published:** 2022-07-19

**Authors:** Harshita Singh, Pallavi Singh, Shashi Bhushan Agrawal, Madhoolika Agrawal

**Affiliations:** Laboratory of Air Pollution and Global Climate Change, Department of Botany, Institute of Science, Banaras Hindu University, Varanasi, India

**Keywords:** particulate matter, tree species, nutrients, stoichiometry, allocation, re-translocation efficiency

## Abstract

The ramifications of different concentrations of foliar particulate matter on the physiology, nutrient stoichiometry, allocation pattern, and their corresponding re-translocation rates were investigated for evergreen (*Mangifera indica* and *Psidium guajava*), semi-evergreen (*Ficus religiosa* and *Azadirachta indica*), and deciduous (*Dalbergia sissoo*) tree species in a simulation experiment over an exposure period of 2 years. Physiological parameters (P_n_, g_s_, Ci, E, and WUE), nutrient stoichiometry (C: N) in different plant parts, and their allocation pattern for five macro- (C, N, K, Mg, Ca) and five (Zn, Ni, Mn, Cu, Fe) micro-elements at two different concentrations of particulate matter (ambient and elevated) with respect to control (no particulate load) were assessed. Significant differences in nutrient concentrations and their re-translocation rates were observed between the treatments in evergreen species compared to deciduous species. The photosynthetic rate significantly declined with an increase in foliar deposition of particulate matter. Higher variations in C, N, K, Mg, and Zn levels were found compared to other elements under particulate matter stress and the ratio of C/N showed a slight decline in mature leaves except in deciduous tree species. The nutrient stoichiometry revealed that the deciduous species were more tolerant whereas the re-translocation efficiency was maximum for the semi-evergreen tree species. The nutrient allocation was found greater in foliage compared to branch in evergreen and was opposite in semi-evergreen and deciduous tree species. The element re-translocation rate indicated an inconsistent behavior in nutrient recycling under the particulate matter load depending upon the tree species. The study entrenched a critical change in nutrient re-translocation and allocation pattern under the particulate stress in different parts of the tree, suggesting a novel approach for screening the tree species for sustainable plantation and planning of urban areas.

## Introduction

The occurrence of particulate matter (PM) is ubiquitous in the tropospheric layer exerting an adverse impact on the climate and environment around the world. At the local level, PM can intensify the effects of greenhouse gases by scattering or absorbing incoming solar radiation that emitted back from the surface of the earth, thereby contributing to the global climate change (Buseck and Pósfai, 1999). Reduction in the concentration of PM particularly PM_2.5_ is of utmost importance as it has been reported to severely affect human health and the environment (Mukherjee and Agrawal, 2017). In urban areas, vehicles and industries are major anthropogenic sources of particulate emissions, despite their identification, halting these sources is neither economically feasible nor functionally possible. One of the best natural methods for the curtailment of PM is to increase urban greening which will provide a surface for the adherence of PM (Escobedo and Nowak, 2009). Adverse effects of PM have already been established on human health (Anderson et al., 2012; Patra et al., 2016; Hassan et al., 2021), but impacts on the plants especially trees, which are extensively used for the mitigation of PM, are still unexplored. Most of the studies relating PM with tree species either provide insight into their capture potential (Chen et al., 2017) depending upon their leaf characteristics (micromorphology, orientation, etc.) (Liu et al., 2018) or screen them as tolerant or sensitive based on their air pollution tolerance index (APTI) and related parameters (pH, relative water content, ascorbic acid, and total chlorophyll) (Sgrigna et al., 2020; Zhang et al., 2020).

PM deposition on the foliage of trees imposes its adverse impacts primarily through the clogging of the stomatal aperture thereby increasing leaf temperature which in turn affects the overall development of the plant (Popek et al., 2018). The layer of PM covering the foliage affects the foliar physiology by acting as a screen between the surface of the leaf and its ambiance. Thereby, blocking the environmental cues. In similar ways, it also hampers the amount of PAR received by the foliage (Mina et al., 2018). There are studies focusing on the nutrient status of plants subjected to various air pollutants (Cao et al., 2016; Shi et al., 2016b; Braun et al., 2020; Piao et al., 2020), but none of the studies have focused on the nutrient-related parameters of the trees under PM stress. For assessing the nutrient status of a plant, it is important to determine the nutrient allocation pattern, along with the determination of nutrient stoichiometry, and ultimately establish the nutrient re-translocation efficiency (NURE) under the applied stress. The allocation of nutrients in plant parts is indicative of plant resource uptake and utilization (Zhao et al., 2020). Leaf stoichiometry is an important determinant of plant composition and nutrient limitation over a period of time (Lanuza et al., 2019). One of the most essential processes to trace nutrient dynamics of tree species is analyzing the NURE. It refers to the quantity of nutrients reabsorbed from the organs of the plant which are showing signs/symptoms of senescence and utilized in the development of new plant parts (Shi et al., 2017).

The present study assesses the nutrient status and unravels the details of nutrient allocation of the dominant tree species employed for urban plantation in the Indo-Gangetic plains under a gradient of PM as a stress factor.

## Materials and Methods

### Experimental Details

**FIGURE 1:**
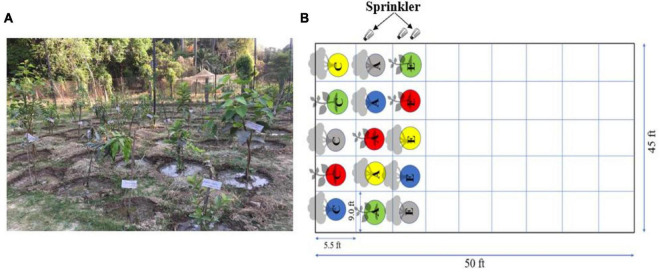
Representation of experimental setup: **(A)** the experimental field with test plant, **(B)** schematic representation of the field. The design is a split plot with a random plantation. The abbreviations for treatments are: C, control PM; A, ambient PM; and E, elevated PM.

The simulation experiment was performed for 2 consecutive years from November 2019 to November 2021 at the Botanical Garden of Banaras Hindu University, Varanasi, Uttar Pradesh, India. The 25° 81_N and 83° 1_E represent the coordinates of the experimental site situated on the eastern Gangetic plains of India. The details of the experimental setup show field dimensions along with the distance between individual tree species (Figure 1). The field design was a split plot with random plantation of tree species and treatment strategy.

### Meteorological Parameters

**FIGURE 2:**
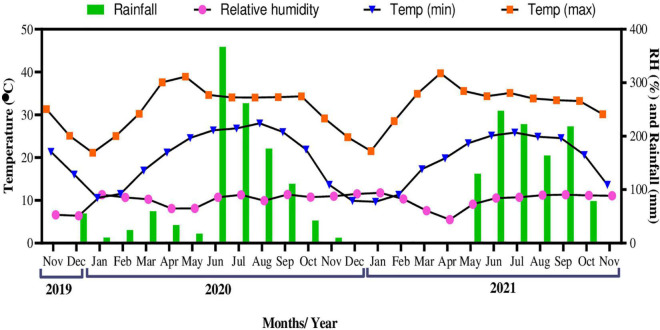
Variations in daily temperature (°C), relative humidity (%), and total rainfall (mm) during the experimental period.

The meteorological data including daily maximum and minimum temperature, total rainfall, and relative humidity, for the experimental period were obtained from the Indian Meteorological Division (IMD) of Banaras Hindu University, Varanasi, India. The mean maximum temperature recorded from November 2019 to November 2021 was 39.72°C in April 2021, while the mean minimum temperature was 9.66°C in January 2021 (Figure 2). The highest temperature was recorded to be 45.9°C on May 27, 2020, and the lowest to be 4.6°C on January 31, 2021. Mean relative humidity (RH) for the study period ranged from 51.10 to 91.03%. Maximum total rainfall was recorded to be 366.9 mm in June 2020.

### Plant Material and Experimental Setup

Dominant trees of the Indo-Gangetic plains belonging to different functional types (evergreen and deciduous) with different leaf forms (simple and compound) (Table 1) were subjected to two different loads of PM *viz*. ambient (APM) and elevated (EPM) with a parallel control (CPM) setup according to the species-specific capture potential of the PM. The present study focused on the nutrient allocation pattern and stoichiometry of different plant parts and re-translocation efficiency of the studied elements in various tree functional types (two evergreen, two semi-evergreen, and one deciduous tree species). One-year-old saplings were purchased from the Horticulture Department of Banaras Hindu University, Varanasi, and were transplanted in the field of dimension 45 × 50 sq. ft. randomly optimizing the distance between the two saplings. A controlled release formulation was applied to the field soil for fertilization containing 13.4% organic carbon, 1.61% nitrogen, 1.02% phosphorus, 0.73% potassium, 7.61% calcium, 0.568% magnesium, 0.158% sodium, 0.110% zinc, and 0.0048% copper. Tap water was used for maintaining the optimum irrigation during the experimental period.

**TABLE 1:** The details of the trees under study.

S.No	Tree species	Family	Functional type	Leaf form	Leaf/Leaflet shape
1.	*Mangifera indica* Linn.	Anacardiaceae	Evergreen	Simple	Lanceolate
2.	*Psidium guajava* Linn.	Myrtaceae	Evergreen	Simple	Obovate
3.	*Ficus religiosa* Linn.	Moraceae	Semi-evergreen	Simple	Cordate
4.	*Azadirachta indica* Juss.	Meliaceae	Semi-evergreen	Compound	Falcate
5.	*Dalbergia sissoo* Roxb.	Fabaceae	Deciduous	Compound	Orbiculate

The PM for simulating the stress condition in the experiment was collected passively from the foliage of the trees in commercial area. The PM adhered on the leaves was washed with distilled water and the water was evaporated on a hot plate (80°C). The PM collected was used for the simulation experiment. The species-specific ambient load was determined using the methodology for deposition determination by Singh et al., 2021 mentioned in Table 2. Dust simulation was manually done with the help of a sprinkler (which was standardized according to its pore size, i.e., the PM expelled from it was quantified and the dose was given accordingly) using water as a surfactant for the adherence of dust on the leaf surface of each plant and the dusting was repeated after every 30 days interval. The EPM was double that of APM and was simulated similarly to APM.

**TABLE 2:** Species-specific PM load was applied during the experimental period.

S. No.	Tree species	Ambient load (g cm^–2^)	Elevated load (g cm^–2^)	Exposure gap (Days)
1.	*Mangifera indica*	0.124	0.248	30
2.	*Psidium guajava*	0.190	0.380	30
3.	*Ficus religiosa*	0.182	0.365	30
4.	*Azadirachta indica*	0.024	0.047	30
5.	*Dalbergia sissoo*	0.008	0.016	30

### Plant Sampling and Analysis

Nine plants of each species were assessed for physiological and nutrient analyses. Nine replicates of each species were considered for physiological measurement, that is, net photosynthesis (P_n_), stomatal conductance (g_s_), internal CO_2_ concentration (C_i_), and transpiration (E) using LICOR photosynthetic system (Model 6200, LICOR, Lincoln, United States). Water Use Efficiency (WUE) was calculated as the ratio of photosynthetic rate to the rate of transpiration. The instrument was calibrated with a known source of CO_2_ (509 ppm) before recording the measurements on a clear sky day. The data collection for the photosynthetic parameters was done between 8:00 and 10:00 h. This was collected from a fully expanded leaf, fourth from the top of the secondary branch of each treatment. The photosynthetically active radiation ranged between 1,100 and 1,200 μmol m^–2^ s^–1^.

The sampling for assessment of nutrient-related parameters was done at the end of the study period. The samples were collected in December 2021 after an exposure period of 2 years. Three mature leaves, senesced leaves, and branch samples from each treatment were randomly collected. The fresh leaves and branches were oven-dried at 70°C to constant weight. The dried samples were grounded in an automatic grinder and passed through a 0.15 mm sieve. The determination of C and N was done by an automatic CHNS analyzer (Euro EA Elemental Analyzer, EURO VECTOR Instruments). Apart from these, K, Mg, Ca, Zn, Ni, Mn, Cu, and Fe were quantified by atomic absorption spectrometer (Model Analyst 800, Perkin-Elmer and Norwalk, United States.) after digesting air-dried plant samples (0.1 g) in 10 mL di-acid (nitric acid:perchloric acid = 9:4) for total metal content (Gaidajis, 2003).

Repeated analyses of samples were conducted against the National Institute of Standards and Technology Standard Reference Material (NBS SRM-1570) for all the metals except C and N for which sulphanilamide OAS [Euro Vector S.r.l., Via F.lli Cuzio, Pavia (PV), Italy] was used to ensure the precision and accuracy of the analysis. To re-calibrate the instrument, blank and drift standards (Sisco Research Laboratories Pvt. Ltd., India) were run after every five sample runs. The results were found to be within 2% of the certified value. For different determinations and precision of analysis, the coefficients of variance of replicate analysis were determined. The variances were found to be fewer than 10%.

The relevant calculations for the determination of nutrient reabsorption efficiency (NURE) were done following the formula of Aerts (1996):


NURE=

$$\left(N\,u\,t\,r\,i\,e\,n\,t\,m\,a\,t\,u\,r\,e\,l\,e\,a\,f-\frac{N\,u\,t\,r\,i\,e\,n\,t\,s\,e\,n\,e\,s\,c\,e\,d\,l\,e\,a\,f}{N\,u\,t\,r\,i\,e\,n\,t\,m\,a\,t\,u\,r\,e\,l\,e\,a\,f}\right)\times 100$$

### Statistical Analysis

The statistical analyses of the experimental data were performed by the IBM SPSS (Statistics 21.0) software. Data were first cohered for normality using the Shapiro–Wilk test and homogeneity of variance by Levene’s test. After meeting this analysis, the data were interpreted for analysis of variance (ANOVA), the difference among the means of different treatments with the same species and the variations among the means of different trees within the same treatments for each species were assessed *via* one-way ANOVA and least significant difference (LSD) *post-hoc* test, respectively. F-ratios were determined for various nutrients in the considered tree species, and treatment and parts by three-way ANOVA. The F-ratios for the other parameters apart from the nutrients were determined for tree species and treatment by two-way ANOVA. A significance level of 0.05 was the basis of statistical significance. The correlation coefficients were plotted using R 4.1.2 (R Core Development Team, 2021).

## Results

### Physiological Parameters

**FIGURE 3:**
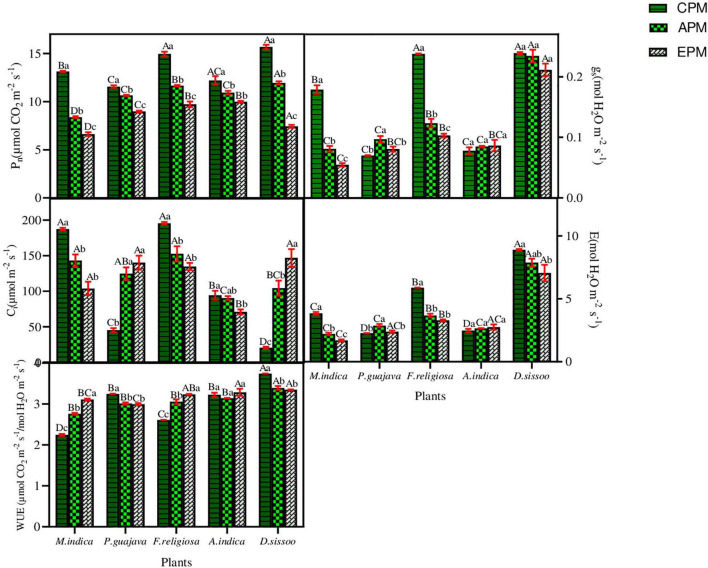
The photosynthetic parameters of different tree species under different PM treatments. Different letters in uppercase indicate a significant difference between PM treatments for different tree species (*p* < 0.05) and different lowercase letters indicate a significant difference between the treatments of a species (*p* < 0.05).

The applied PM load significantly reduced the assimilative rate in all the species under the study (Figure 3). The evergreen trees with simple leaves showed a greater reduction between treatments which was highest in *M. indica*, that is, 36.69% in APM and 49.55% in EPM, and lowest in *P. guajava*, which was 7.93% in APM and 22.40% in EPM. It was reduced by 10.35% in APM and 18.14% in EPM of *A*. *indica* which is semi-evergreen with compound leaves, while it was reduced by 23.86% in APM and 52.60% in EPM in *D. sissoo*, which is a deciduous tree with compound leaves. ANOVA test showed that both the treatments significantly affected all the physiological parameters of all the tree species individually and in combination (Table 3).

**TABLE 3:** F-ratios and level of significance of two-way ANOVA test for various parameters of the test tress species.

Nutrients	Tree species	Treatment	Tree X treatment
Pn	147.644***	615.452***	81.939***
gs	36.115***	12.266***	5.047**
Ci	87.836***	12.063***	71.415***
E	523.013***	31.796***	22.236***
WUE	171.416***	28.127***	47.219***
NURE_C	157.236***	116.286***	97.492***
NURE_N	129.270***	5.421***	179.788***
NURE_K	112.182***	58.089***	13.565***
NURE_Mg	40.313***	10.789***	98.228***
NURE_Ca	9.923***	11.486***	1.688***
NURE_Fe	22.474***	3.231^ns^	3.197*
NURE_Cu	321.554***	20.167***	21.156***
NURE_Mn	259.940***	198.786***	118.329***
NURE_Ni	341.452***	117.204***	77.174***
NURE_Zn	381.969***	182.345***	468.368***

Level of significance: ns, non significant, *p < 0.05, **p < 0.01, ***p < 0.001.

An inconsistency was observed in the g_s_ of the studied species (Figure 3). A significant reduction in the g_s_ was found in *M. indica* with 54.75% in APM and 69.52% in EPM. Similarly, *F. religiosa* showed a decline of 48.15% in APM and 56.77% in EPM. An exception was observed for *P. guajava* which showed a significant increase in g_s_ of 38.48% in APM and 15.31% in EPM. *A. indica* followed a similar increase in g_s_ as of *P. guajava* whereas *D. sissoo* showed an insignificant decline.

The C_i_ concentration was found to decline remarkably in simple leaf species with an exception in *P. guajava* where an increase in C_i_ of 176.36% in APM and 210.84% in EPM was found (Figure 3). *A. indica* with compound leaf comparably showed less reduction in C_i_ of 4.30% in APM and 24.79% in EPM. *D. sissoo* displayed the maximum increase in C_i_ of 412.66% in APM and 622.03% in EPM treatments.

EPM decreased maximally in *M. indica* by 42.60% in APM and 55.88% in EPM and least in *D. sissoo* which was 11.45% in APM and 20.69% in EPM (Figure 3). *P. guajava* and *A. indica* showed an increase in transpiration rate of 37.15% and 6.96% in APM and 44.15% and 11.32% in EPM, respectively.

The WUE increased in *M. indica* and *F. religiosa* whereas decreased in *P.* guajava, *A. indica*, and *D. sissoo* (Figure 3). The increase was maximum in *M. indica* (22.83% in APM and 38.40% in EPM) while the reduction was maximum in *D. sissoo* (9.35% in APM and 10.56% in EPM).

### Allocation Pattern Under Particulate Matter Stress

It was observed that the nutrients considered follow two different patterns of allocation and re-translocation: (i) macroelements and (ii) microelements. Macroelements were further studied under two major divisions: phloem immobile elements (water in-soluble) and phloem mobile (water soluble) elements. Carbon is described separately as it follows both immobile and mobile behaviors. Phloem immobile elements are manganese (Mn) and calcium (Ca) and mobile elements are nitrogen (N), potassium (K), and magnesium (Mg). The micronutrients include copper (Cu), zinc (Zn), iron (Fe), and nickel (Ni).

### In Mature Leaves

**FIGURE 4:**
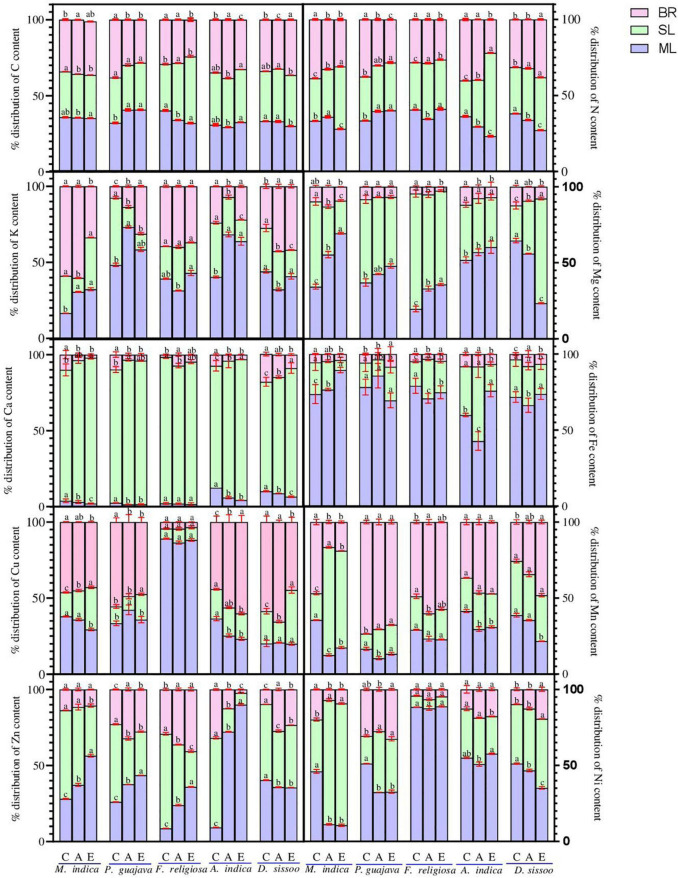
The nutrient allocation pattern of different tree species under different PM treatments. Different lowercase letters indicate a significant difference between the treatments within species (*p* < 0.05). BR, branch; SL, senesced leaf; ML, mature leaf.

The phloem immobile elements namely Mn and Ca showed a similar trend for the mature leaves (Figure 4). They displayed an increase in APM which ranged between 13.3 and 31.0% for Mn and 1.6% and 6.6% for Ca. The increase was maximum in *A. indica* for Mn and *D. sissoo* for Ca. A decrease was observed in the allocation under EPM which ranged between 8.3 and 64.8% for Mn and 9.2% and 51.8% for Ca. The decrease was maximum in *M. indica* for Mn and *A. indica* for Ca. For the phloem mobile elements, namely N, K, and Mg, an increase was observed in APM which ranged between 23.2 and 69.3%. The increase was minimum for N in *A. indica* and maximum for Mg in *M. indica*. EPM showed an inconsistent trend where a reduction (11.1–19.9%) was found in *F. religiosa*, *A. indica*, and *D. sissoo* for N, in *F. religiosa* for K, and in *D. sissoo* for Mg, whereas all others showed increases. The allocation of C displayed a marked decline with increasing PM load (Figure 4). It decreased between 0.5 and 15.5% under APM and 1.7% and 20.5% under EPM with an exception of *P. guajava*, where an increase of 26% was observed in both the treatments.

The microelements Cu, Zn, Ni, and Fe, showed a consistent increase in the allocation pattern which ranged between 10.7 and 90% (Figure 4). This was highest in *M. indica* for Ni and lowest in *A. indica* for Zn. The EPM showed a marked decline in most of the microelements which ranged from 1 to 75.5% and was maximum in *M. indica* and minimum in *F. religiosa* for Ni.

### In Senesced Leaves

The immobile elements showed an increase under APM ranging between 19 and 95.9% being minimum in *P. guajava* for Mn and *M. indica* for Ca (Figure 4). Under EPM, there was a marked increase in all except *F. religiosa* for both Mn and Ca and *D. sissoo* for Mn. The increase under EPM ranged between 6.3% in *D. sissoo* for Ca and 304.6% in *M. indica* for Mn. For the allocation of carbon, no definite trend was found under both the treatments. Carbon allocation increased in *F. religiosa* and *D. sissoo* under APM while decreased in all other tree species. Under EPM treatment, an increase was observed in C allocation from 1.5 to 43.6% except in *M. indica* where the allocation decreased by 5.3%.

The phloem mobile elements showed an increase in allocation pattern under APM which ranged between 10.2 and 68.8% (Figure 4). It was highest in *D. sissoo* for Mg and lowest in *P. guajava* for K. Under EPM, an increase was observed in all species for N whereas others showed a marked decline ranging between 1.9 and 70%, being maximum in *A. indica* for Mg and minimum for *P*. *guajava* for K. *F. religiosa* was an exception for K and *D. sissoo* for Mg which showed increment under EPM. For microelements, an increase was found under APM which ranged between 6.5 and 80% (Figure 4). It was found to be maximum in *M. indica* and minimum in *F. religiosa* for Ni. Under EPM, a reduction was observed in most microelements which ranged from 4.76 to 73.9% being highest in *A. indica* for Cu and lowest for Zn.

### In Branches

The allocation pattern of the immobile elements consistently increased in all species under APM ranging from 18.9 to 96% and the increase was maximum in *M. indica* for Ca and minimum for Mn (Figure 4). Under EPM, most of the species showed an increase in allocation which ranged between 8.3 and 34% except Mn in *M. indica* and *P. guajava* and Ca in *F. religiosa*.

All phloem mobile elements under APM showed an increased allocation ranging between 2.7 and 38% (Figure 4). It was highest in *D. sissoo* for N and lowest in *F. religiosa* for Mg. Both increase and decrease in allocation were observed under EPM (Figure 4). The increase ranged between 1.4 and 80% which was maximum in *P. guajava* for K and minimum in *F. religiosa* for N. The decline ranged between 1.2 and 70.2% being highest for *A. indica* for K and lowest for N.

The microelements showed an increment in all the species under APM with a range of 2.3% to 60%, where *A. indica* for Cu showed a maximum increase, while the same showed a minimum for Zn. Most of the species under EPM showed an increase ranging between 1.3 and 182.4% and was maximum for Zn in *D. sissoo* and minimum for Fe in *A. indica*.

### Nutrient Re-translocation Efficiency Under the Particulate Matter Stress

**FIGURE 5:**
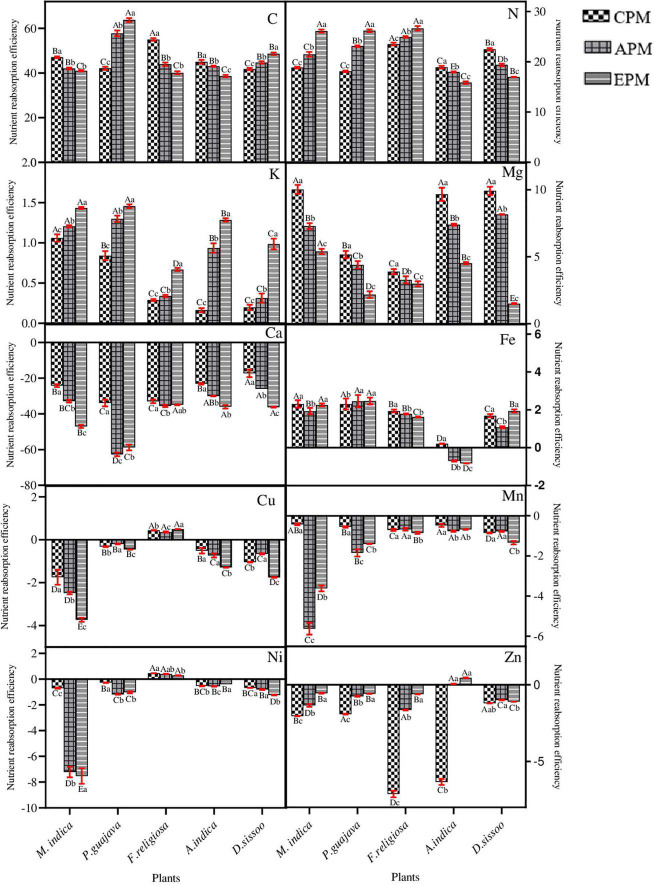
The nutrient re-translocation efficiency of different tree species under different PM treatments. Different letters in uppercase indicate a significant difference between PM treatments for different trees (*p* < 0.05) and different lowercase letters indicate a significant difference between the treatment with species (*p* < 0.05).

Among the 10 nutrients under consideration, 4 showed a consistent positive re-translocation (C, N, K, Mg) while 6 were negatively re-translocated (Ca, Fe, Cu, Mn, Ni, Zn) under the PM stress (Figure 5). The phloem immobile elements possessed negative NURE which decreased with the PM load for both Ca and Mn. Among the mobile elements, K showed an increase in NURE in all whereas Mg showed a marked decline with increasing PM load (Figure 5). A decrease in NURE with PM load was found for *A. indica* and *D. sissoo* while others showed a significant increase for N. For C, NURE showed a positive value which decreased with increasing PM load except in *P. guajava* and *D. sissoo* where the value increased in PM-treated plants.

Among the microelements, Cu and Ni showed negative NURE which decreased with increasing PM for all except *F. religiosa*. Zn also showed negative NURE for all the species but the allocation was significantly increased with PM load. Fe showed positive NURE for all except *A. indica*, where the PM-treated species showed significantly negative NURE.

All the trees consistently showed positive NURE for C which decreased with PM load in *M. indica, F. religiosa*, and *A. indica* whereas increased in *P. guajava* and *D. sissoo.* ANOVA test showed that the treatments significantly affected the nutrients in all species except for Fe where the effect was non-significant (Table 4).

**TABLE 4:** F-ratios and level of significance of three-way ANOVA test for various nutrients of the test tree species.

Nutrients	Tree species	Treatment	Part	Tree X treatment	Tree X part	Treatment X part	Tree X treatment X part
C	0.409^ns^	0.218^ns^	322.156***	0.539^ns^	338.293***	490.895***	96.11***
N	67.995***	0.611^ns^	438.438***	67.995***	475.781***	295.035***	632.502***
K	0.608^ns^	0.578^ns^	109.807***	3.589**	50.686***	29.191***	13.251***
Mg	0.807^ns^	24.495***	650.451***	82.663***	42.129***	20.699***	29.831***
Ca	7.699***	0.028^ns^	143.199***	0.396^ns^	24.010***	5.079**	4.254***
Fe	0.124^ns^	0.793^ns^	106.703***	0.034^ns^	11.066***	20.273***	4.852***
Cu	97.157***	81.752***	510.667***	24.347***	473.084***	8.399***	19.197***
Mn	0.261***	2.103^ns^	318.519***	0.663^ns^	130.568***	2.258^ns^	25.946***
Zn	0.142***	67.995***	36.327***	0.386^ns^	115.104***	108.318***	599.650***
Ni	0.310***	0.172^ns^	76.451***	0.267^ns^	296.247***	65.489***	221.140***

Level of significance: ns, non significant; *p < 0.05, **p < 0.01, ***p < 0.001.

### Nutrient Stoichiometry Under the Particulate Matter Stress

**FIGURE 6:**
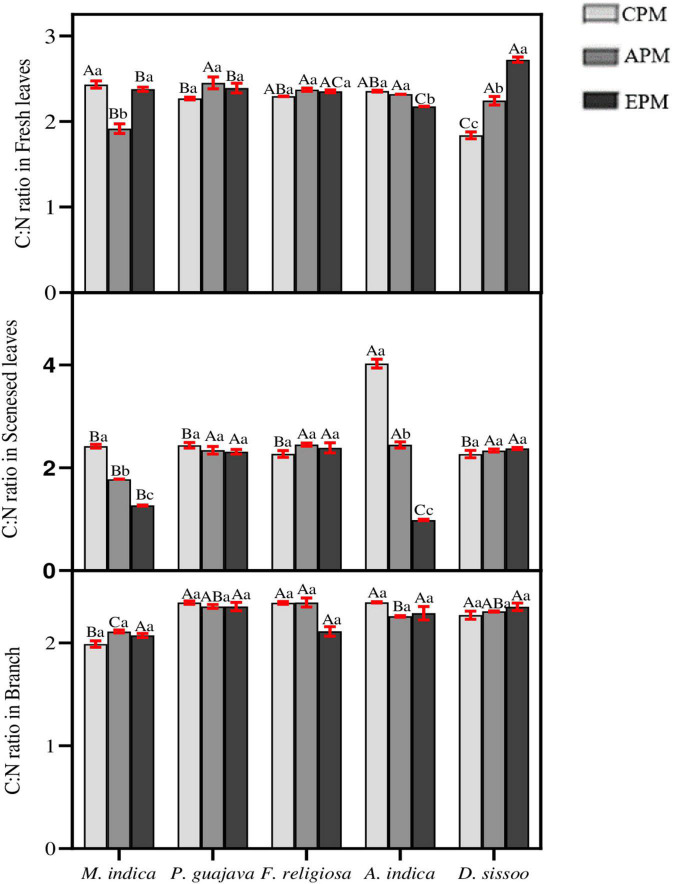
The nutrient stoichiometry of C and N for different tree species under different PM treatments. Different letters in uppercase indicate a significant difference between PM treatments for different trees (*p* < 0.05) and different lowercase letters indicate a significant difference between the treatment with species (*p* < 0.05).

Nutrient stoichiometry between C and N was analyzed between different parts of the species under study (Figure 6). In mature leaves, an inconsistency was observed among the trees for C:N ratio. It significantly increased in *D. sissoo* whereas a decrease was found in *M. indica* and *A. indica*. In the senesced leaves, a significant decrease was observed in *M. indica* and *A. indica* whereas all others remained unaffected. No differences were found between the C:N ratio of branches in all the tree species under PM treatments.

### Principal Component Analysis

**FIGURE 7:**
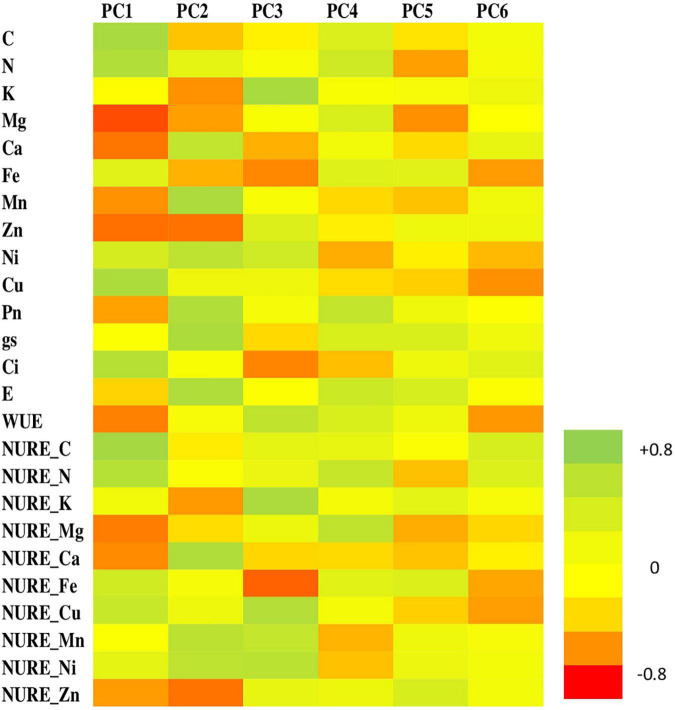
Heat map of six principal components (PCs) showing respective loadings of the parameters during the experimental period.

The PCA conducted for parameters under study showed 86.17% of total variance, explained by six PCs (Figure 7). In the course of experiment, the primary component (PC1, eigen value = 6.350, variance = 25.40%) established C, N, NURE-C, and NURE-N as dominant variables. PC2 (eigen value = 5.543, variance = 22.17%) showed Ca, P_*n*_, g_*s*_, and E with higher loading values whereas PC3 (eigen value = 4.220, variance = 16.88%) unveiled Ni, Cu, NURE-Cu, NURE-Mn, and NURE-Ni as the variables with higher loading. PC4 (eigen value = 2.520, variance = 10.08%) showed K, Ca, Zn, NURE-K, NURE-Ca, and NURE-Zn as the higher loading variables. PC5 (eigen value = 1.535, variance = 6.14%) demonstrated Mg, Ci, WUE, and NURE-Mg as the dominant variables, while Fe, NURE-Fe, and NURE-Mn found to be loaded maximally on PC6 (eigen value = 1.374, variance = 5.49%).

## Discussion

The amount of nutrients required to be re-translocated and the rate of re-translocation is influenced by plant species, mobility of nutrients, and environmental stress conditions (Hagen-Thorn et al., 2004). PM deposition on the foliage of tree species varying in their functional types (evergreen or deciduous) and its consequences on nutrient status, nutrient allocation, and nutrient re-translocation efficiency were investigated in the present study. The physiological performance is assessed in terms of the plant’s photosynthetic capacity. In the present study, Pn showed a marked decline in all the tree species under both APM and EPM and the decrease was more pronounced in EPM. In terms of g_*s*_, a significant decrease was observed in *M. indica* and *F. religiosa* whereas an increase was observed in *P. guajava* which can be attributed to its hypostomatic condition as reported in the study of Shiva et al. (2017). A drop in Ci was observed for *M. indica*, *F. religiosa*, and *A. indica* while *P. guajava* and *D. sissoo* showed a significant increase which can be attributed to their corresponding stomatal behavior as previously reported by Jaiswal et al. (2021). A significant reduction was observed in EPM for all the tree species except *P. guajava* and *D. sissoo* which is also supported by the corresponding change in g_*s*_ as previously reported in the study of Mahmood et al. (2016). WUE is in line with the E of the studied tree species where a marked increase was observed in WUE with a decrease in EPM.

In the plants, nutrients are required for maintaining the redox balance and changes caused in them due to stress conditions may lead to an unfavorable milieu in the plants (Tripathi et al., 2018). It is already established that the factors promoting plant growth will positively affect the re-translocation efficiency and vice versa (Nambiar and Fife, 1991). The results of this revealed distinct patterns of nutrient allocation and re-translocation in trees belonging to different functional types. The phloem-immobile elements, namely, Ca and Mn, primarily remain in senesced leaves leading to their deficiencies in mature leaves thereby anticipating negative values on reabsorption rates (Shi et al., 2016a). This is in line with our study where Ca displayed a continuous accumulation in senesced leaves and a negative NURE while the accumulation of Mn was found to be greater in the branches to prevent its phytotoxic impacts in mature leaves (Farahat and Linderholm, 2015). Due to the oxidative stress induced in tree species under PM treatment, it required Mn for the increased function of Superoxide Dismutase (SOD) (Alscher et al., 2002) which is the sole reason for its enhancement in treated plants.

**FIGURE 8:**
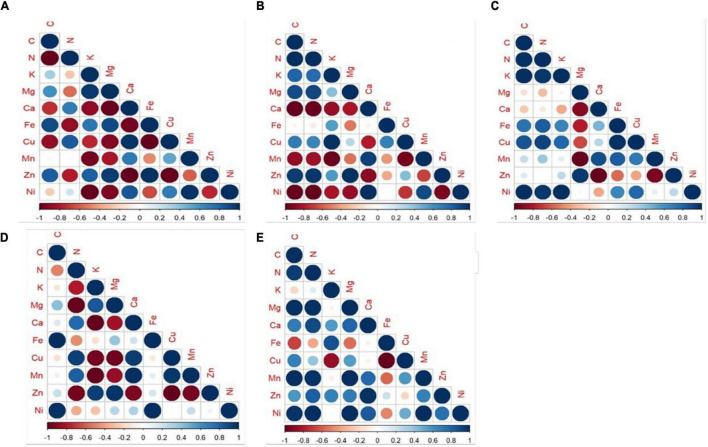
Correlation between the nutrients under study in mature leaves subjected to different treatments of PM- **(A)** *M. indica*, **(B)** *P. guajava*, **(C)** *F. religiosa*, **(D)** *A. indica*, **(E)** *D. sissoo*. Blue-large dots showed maximum positive correlation and red-large dots showed maximum negative correlation with a gradient of color and size showing in between correlation values.

The pattern of re-translocation observed for water-soluble elements N, K, and Mg that are phloem-mobile and should be re-translocated from old to younger leaves anticipates a positive value on reabsorption rate (Maillard et al., 2015). This is consistent with our study, where N, K, and Mg were found to be readily translocated from senesced to mature leaves and thereby anticipated positive values on the reabsorption rates of all the species. In spite of positive NURE, the efficiency was greater for PM-stressed plants in *M. indica, P. guajava*, and *F. religiosa* compared to CPM. The increase in NURE of N might be due to increased proteolysis under applied stress which would in turn increase the availability of N for re-translocation to actively growing parts of the trees (Temple and Riechers, 1995). In general, Pn is correlated with the N concentration which is displayed by *A. indica* and *D. sissoo* (Poorter and Evans, 1998). The major function of K in plants is its involvement in osmoregulation. It can be inferred from the study that PM-induced osmotic stress imposed an increase in K concentration as observed consistently in the PM-treated tree species compared to CPM, thereby favoring physiological processes important for the plants (Hsiao and Läuchli, 1986). It can also be correlated with the transpiration and WUE which decreased in the subsequent PM treatment where K concentration increased (Läuchli, 1984). Hence, the plants water economy depends on the K status of plants (Hsiao and Läuchli, 1986) and retards water loss through transpiration. Mg invariably decreases with an increase in PM load which can be attributed to the antagonistic effect of K over Mg (Ohno and Grunes, 1985). K and Mg exhibited functional synergism in various metabolic processes and Mg can partially replace K in some functions (Xie et al., 2021). Both are found to be negatively correlated in the present study (Figure 8).

The micronutrients considered in this study showed negative NURE because of their low mobility and phytotoxic behavior (Farahat and Linderholm, 2015) except Fe. In spite of their negative NURE, Cu and Zn along with Fe (showed positive NURE) showed lesser accumulation in senesced leaves under increasing PM load which might be due to the oxidative stress induced by PM which in turn increased the activity of different groups of SOD, namely, Fe SOD and Cu/Zn SOD (Alscher et al., 2002), which are common elements required for the functioning of SOD. Fe SOD is mainly located in chloroplast and Cu/Zn SOD in the cytosol, chloroplasts, and peroxisomes. The concentrations of Fe, Cu, and Zn were found to decrease in senesced leaves as they are required for the enhanced activity of SOD under PM-induced oxidative stress. Another micronutrient Ni showed negative NURE which might be an effort to protect the tree species from the phytotoxic effect of Ni (Farahat and Linderholm, 2015).

The carbon showed differential allocation and NURE patterns. In *M. indica, F. religiosa*, and *A. indica*, the NURE decreased with an increase in PM load which can be correlated with a decrease in g_*s*_, which in turn reduces the atmospheric uptake of carbon (Sulman et al., 2016). In *P. guajava* and *D. sissoo*, an increase in NURE was observed with increasing PM concentration which might be due to their hypostomatic leaf, thereby facilitating the uptake of carbon which is reflected in the insignificant changes in g_*s*_.

The re-translocation efficiency was found to be affected more in evergreen trees with increasing PM load compared to semi-evergreen or deciduous tree species under study. The evergreen trees have a simple leaf type which provided greater surface area for the adherence of PM. Thus, the higher the loading of PM, the greater the impact observed. To meet their higher demand for assimilating translocation, evergreen trees with longer leaf lifespan need to invest more in phloem loading and export apparatus (Bazzaz and Grace, 1997; Givnish, 2002). Thereby increasing the allocation of nutrients more to branches than in leaves as seen in our study. On the contrary, deciduous trees evolved with a short leaf lifespan allocate more nutrients to the leaf as they have a requirement to maximize their photosynthetic activity to utilize the light accessibility during the shorter growing period (Yan et al., 2016). This is in line with the results obtained in our study. Based on the correlation between different elements, it is inferred that the evergreen trees showed more correlation among studied elements compared to the deciduous trees, whereas most of the elements displayed a non-correlating relationship in semi-evergreen tree species (Figure 8). The uptake of nutrients depends on the specific transporter present on the plasma membrane of the cells (Barzana et al., 2021). The differences in the correlation pattern of nutrients observed in the present study might be due to the change in nutrient transporters (number or nature) under the applied stress of PM.

Our results suggest that both APM and EPM imposed significant impacts on the nutrient status of the tree species. About the evergreen tree species, *Psidium guajava* is relatively more tolerant under the applied stress. In the semi-evergreen tree species, *F. religiosa* showed a better response and can be considered efficient in enduring the PM stress. Among all the studied species, *F*. *religiosa* is marked as an efficient remobilizer due to its ability to remobilize 7 nutrients efficiently out of the studied 10 nutrients with decreasing NURE for most of the nutrients under increasing PM load. Thus, *P. guajava* and *F. religiosa*, owing to their better tolerance, can be used extensively for plantation and green belt development in and around urban areas.

## Data Availability Statement

The original contributions presented in this study are included in the article/supplementary material, further inquiries can be directed to the corresponding author.

## Author Contributions

HS planned and conducted the experiment, did statistical analysis, and data curation. HS and PS wrote the manuscript. MA and SA visualized, investigated, and edited the manuscript. MA supervised the work. All authors contributed to the article and approved the submitted version.

## Conflict of Interest

The authors declare that the research was conducted in the absence of any commercial or financial relationships that could be construed as a potential conflict of interest.

## Publisher’s Note

All claims expressed in this article are solely those of the authors and do not necessarily represent those of their affiliated organizations, or those of the publisher, the editors and the reviewers. Any product that may be evaluated in this article, or claim that may be made by its manufacturer, is not guaranteed or endorsed by the publisher.

## References

[B1] Aerts R. (1996). Nutrient resorption from senescing leaves of perennials: are there general patterns? *J. Ecol.* 84, 597–608. 10.2307/2261481

[B2] Alscher R. G. Erturk N. Heath L. S. (2002). Role of superoxide dismutases (SODs) in controlling oxidative stress in plants. *J. Exp. Bot.* 53 1331–1341. 10.1093/jexbot/53.372.1331 11997379

[B3] Anderson J. O. Thundiyil J. G. Stolbach A. (2012). Clearing the air: a review of the effects of particulate matter air pollution on human health. *J. Med. Toxicol.* 8 166–175. 10.1007/s13181-011-0203-1 22194192 PMC3550231

[B4] Barzana G. Rios J. J. Lopez-Zaplana A. Nicolas-Espinosa J. Yepes-Molina L. Garcia-Ibañez P. (2021). Interrelations of nutrient and water transporters in plants under abiotic stress. *Physiol. Plant.* 171 595–619. 10.1111/l.13206 32909634

[B5] Bazzaz F. A. Grace J. (eds) (1997). *Plant Resource Allocation.* Amsterdam: Elsevier.

[B6] Braun S. Schindler C. Rihm B. (2020). Foliar nutrient concentrations of European beech in Switzerland: relations with nitrogen deposition, ozone, climate and soil chemistry. *Front. For. Glob. Change* 3:33. 10.3389/ffgc.2020.00033

[B7] Buseck P. R. Pósfai M. (1999). Airborne minerals and related aerosol particles: effects on climate and the environment. *Proc. Natl. Acad. Sci.* 96 3372–3379. 10.1073/pnas.96.7.3372 10097046 PMC34277

[B8] Cao J. Shang H. Chen Z. Tian Y. Yu H. (2016). Effects of elevated ozone on stoichiometry and nutrient pools of Phoebe Bournei (Hemsl.) Yang and Phoebe Zhennan S. Lee et FN Wei seedlings in subtropical China. *Forests* 7:78. 10.3390/f7040078

[B9] Chen L. Liu C. Zhang L. Zou R. Zhang Z. (2017). Variation in tree species ability to capture and retain airborne fine particulate matter (PM2.5). *Sci. Rep.* 7:3206. 10.1038/s41598-017-03360-1 28600533 PMC5466687

[B10] Escobedo F. J. Nowak D. J. (2009). Spatial heterogeneity and air pollution removal by an urban forest. *Landsc. Urban Plan.* 90 102–110. 10.1016/j.landurbplan.2008.10.021

[B11] Farahat E. Linderholm H. W. (2015). Nutrient resorption efficiency and proficiency in economic wood trees irrigated by treated wastewater in desert planted forests. *Agric. Water Manag.* 155 67–75. 10.1016/j.agwat.2015.03.008

[B12] Gaidajis G. (2003). Ambient concentrations of total suspended particulate matter and its elemental constituents at the wider area of the mining facilities of TVX Hellas in Chalkidiki, Greece. *J. Environ. Sci. Health A Tox. Hazard. Subst. Environ. Eng.* 38 2509–2520. 10.1081/ESE-120024443 14533919

[B13] Givnish T. J. (2002). Adaptive significance of evergreen vs. deciduous leaves: solving the triple paradox. *Silva Fenn.* 36 703–743.

[B14] Hagen-Thorn A. Armolaitis K. Callesen I. Stjernquist I. (2004). Macronutrients in tree stems and foliage: a comparative study of six temperate forest species planted at the same sites. *Ann. For. Sci.* 61 489–498. 10.1051/forest:2004043

[B15] Hassan A. Ilyas S. Z. Agathopoulos S. Hussain S. M. Jalil A. Ahmed S. (2021). Evaluation of adverse effects of particulate matter on human life. *Heliyon* 7:e05968. 10.1016/j.heliyon.2021.e05968 33665396 PMC7903305

[B16] Hsiao T. C Läuchli A. (1986). “Role of potassium in plant-water relations,” in *Advances in Plant Nutrition*, eds Tinker B. Läuchli A. (New York: Praeger). 2 281–312.

[B17] Jaiswal B. Singh S. Agrawal S. B. Agrawal M. (2021). Assessment of physiological, biochemical and yield responses of wheat plants under natural saline and non-saline field conditions. *Physiol. Mol. Biol. Plants* 27 2315–2331. 10.1007/s12298-021-01070-7 34744368 PMC8526689

[B18] Lanuza O. Casanoves F. Delgado D. Van den Meersche K. (2019). Leaf litter stoichiometry affects decomposition rates and nutrient dynamics in tropical forests under restoration in Costa Rica. *Restor. Ecol.* 27 549–558. 10.1111/rec.12893

[B19] Läuchli A. (1984). “Mechanisms of nutrient fluxes at membranes of the root surface and their regulation in the whole plant,” in *Roots, Nutrient and Water Influx, and Plant Growth*, eds Barber S.A. Bouldin D.R. (Hoboken: Wiley). 49 1–25. 10.2134/asaspecpub49.c1

[B20] Liu J. Cao Z. Zou S. Liu H. Hai X. Wang S. (2018). An investigation of the leaf retention capacity, efficiency and mechanism for atmospheric particulate matter of five greening tree species in Beijing. China. *Sci. Total Environ.* 616 417–426. 10.1016/j.scitotenv.2017.10.314 29127795

[B21] Mahmood S. Daur I. Al-Solaimani S. G. Ahmad S. Madkour M. H. Yasir M. (2016). Plant growth promoting rhizobacteria and silicon synergistically enhance salinity tolerance of mung bean. *Front. Plant Sci.* 7:876. 10.3389/fpls.2016.00876 27379151 PMC4911404

[B22] Maillard A. Diquélou S. Billard V. Laîné P. Garnica M. Prudent M. (2015). Leaf mineral nutrient remobilization during leaf senescence and modulation by nutrient deficiency. *Front. Plant Sci.* 6:317. 10.3389/fpls.2015.00317 26029223 PMC4429656

[B23] Mina U. Chandrashekara T. K. Kumar S. N. Meena M. C. Yadav S. Tiwari S. (2018). Impact of particulate matter on basmati rice varieties grown in Indo-Gangetic Plains of India: growth, biochemical, physiological and yield attributes. *Atmos. Environ.* 188 174–184. 10.1016/j.atmosenv.2018.06.015

[B24] Mukherjee A. Agrawal M. (2017). World air particulate matter: sources, distribution and health effects. *Environ. Chem. Lett.* 15 283–309. 10.1007/s10311-017-0611-9

[B25] Nambiar E. S. Fife D. N. (1991). Nutrient retranslocation in temperate conifers. *Tree Physiol.* 9 185–207. 10.1093/treephys/9.1-2.185 14972864

[B26] Ohno T. Grunes D. L. (1985). Potassium-magnesium interactions affecting nutrient uptake by wheat forage. *Soil Sci. Soc. Am. J.* 49 685–690. 10.2136/sssaj1985.03615995004900030032x

[B27] Patra A. K. Gautam S. Kumar P. (2016). Emissions and human health impact of particulate matter from surface mining operation—A review. *Environ. Technol. Innov.* 5 233–249. 10.1016/j.eti.2016.04.002

[B28] Piao H. C. Li S. L. Yan Z. Li C. (2020). Understanding nutrient allocation based on leaf nitrogen isotopes and elemental ratios in the karst region of Southwest China. *Agric. Ecosyst. Environ.* 294:106864. 10.1016/j.agee.2020.106864

[B29] Poorter H. Evans J. R. (1998). Photosynthetic nitrogen-use efficiency of species that differ inherently in specific leaf area. *Oecologia* 116 26–37. 10.1007/s004420050560 28308535

[B30] Popek R. Przybysz A. Gawrońska H. Klamkowski K. Gawroński S. W. (2018). Impact of particulate matter accumulation on the photosynthetic aaratus of roadside woody plants growing in the urban conditions. *Ecotoxicol. Environ. Saf.* 163 56–62. 10.1016/j.ecoenv.2018.07.051 30036757

[B31] Sgrigna G. Baldacchini C. Dreveck S. Cheng Z. Calfapietra C. (2020). Relationships between air particulate matter capture efficiency and leaf traits in twelve tree species from an Italian urban-industrial environment. *Sci. Total Environ.* 718:137310. 10.1016/j.scitotenv.2020.137310 32088481

[B32] Shi C. Eguchi N. Meng F. Watanabe T. Satoh F. Koike T. (2016b). Retranslocation of foliar nutrients of deciduous tree seedlings in different soil condition under free-air O3 enrichment. *iForest* 9:835–841. 10.3832/ifor1889-009 17959540

[B33] Shi C. Kitao M. Agathokleous E. Watanabe M. Tobita H. Yazaki K. I. (2016a). Foliar chemical composition of two oak species grown in a free-air enrichment system with elevated O3 and CO2. *J. Agric. Meteorol.* 72 50–58. 10.2480/agrmet.D-14-00018 15923096

[B34] Shi C. Watanabe T. Koike T. (2017). Leaf stoichiometry of deciduous tree species in different soils exposed to free-air O3 enrichment over two growing seasons. *Environ. Exp. Bot.* 138 148–163. 10.1016/j.envexpbot.2017.03.012

[B35] Shiva B. Nagaraja A. Srivastava M. Goswami A. K. (2017). Determination of Correlation between Stomatal Density and Gas Exchange Traits in Guava. *Int. J. Curr. Microbiol. App. Sci* 6 1328–1334. 10.20546/ijcmas.2017.609.160

[B36] Singh S. Pandey B. Roy L. B. Shekhar S. Singh R. K. (2021). Tree responses to foliar dust deposition and gradient of air pollution around opencast coal mines of Jharia coalfield, India: gas exchange, antioxidative potential and tolerance level. *Environ. Sci. Pollut. Res.* 28 8637–8651. 10.1007/s11356-020-11088-1 33067782

[B37] Sulman B. N. Roman D. T. Yi K. Wang L. Phillips R. P. Novick K. A. (2016). High atmospheric demand for water can limit forest carbon uptake and transpiration as severely as dry soil. *Geophys. Res. Lett.* 43 9686–9695. 10.1002/2016GL069416

[B38] Temple P. J. Riechers G. H. (1995). Nitrogen allocation in ponderosa pine seedlings exposed to interacting ozone and drought stresses. *New Phytol.* 130 97–104. 10.1111/j.1469-8137.1995.tb01819.x

[B39] Tripathi A. M. Klem K. Fischer M. Orság M. Trnka M. Marek M. V. (2018). Water availability influences accumulation and allocation of nutrients and metals in short-rotation poplar plantation. *Biomass Bioenergy* 116 151–160. 10.1016/j.biombioe.2018.06.010

[B40] Xie K. Cakmak I. Wang S. Zhang F. Guo S. (2021). Synergistic and antagonistic interactions between potassium and magnesium in higher plants. *Crop J.* 9 249–256. 10.1016/j.cj.2020.10.005

[B41] Yan Z. Li P. Chen Y. Han W. Fang J. (2016). Nutrient allocation strategies of woody plants: an aroach from the scaling of nitrogen and phosphorus between twig stems and leaves. *Sci Rep.* 6:20099. 10.1038/srep20099 26848020 PMC4742826

[B42] Zhang W. Zhang Y. Gong J. Yang B. Zhang Z. Wang B. (2020). Comparison of the suitability of plant species for greenbelt construction based on particulate matter capture capacity, air pollution tolerance index, and antioxidant system. *Environ. Pollut.* 263:114615. 10.1016/j.envpol.2020.114615

[B43] Zhao N. Yu G. Wang Q. Wang R. Zhang J. Liu C. (2020). Conservative allocation strategy of multiple nutrients among major plant organs: from species to community. *J. Ecol.* 108 267–278. 10.1111/1365-2745.13256

